# Follow-up of breast cancer in primary care vs specialist care: results of an economic evaluation

**DOI:** 10.1038/sj.bjc.6690197

**Published:** 1999-03

**Authors:** E Grunfeld, A Gray, D Mant, P Yudkin, R Adewuyi-Dalton, D Coyle, D Cole, J Stewart, R Fitzpatrick, M Vessey

**Affiliations:** Division of Public Health and Primary Health Care, Institute of Health Sciences, University of Oxford, Oxford, OX3 7LF, UK; Ottawa Regional Cancer Centre, Department of Medicine, University of Ottawa, Ottawa, Ontario, K1H 8L6, Canada; Northamptonshire Centre for Oncology, Northampton General Hospital, Northampton, NN1 5BD, UK; Health Economics Research Centre, Institute of Health Sciences, University of Oxford, Oxford, OX3 7LF, UK; Department of Primary Medical Care, University of Southampton, Southampton, SO1 6ST, UK; Clinical Epidemiology Unit, Loeb Research Institute, University of Ottawa, Ontario, Ottawa, K1Y 4E9, Canada; Princess Margaret Hospital, Swindon, SN1 4JU, UK

**Keywords:** breast cancer, economic evaluation, follow-up care, randomized controlled trial, primary care

## Abstract

A randomized controlled trial (RCT) comparing primary-care-centred follow-up of breast cancer patients with the current standard practice of specialist-centred follow-up showed no increase in delay in diagnosing recurrence, and no increase in anxiety or deterioration in health-related quality of life. An economic evaluation of the two schemes of follow-up was conducted concurrent with the RCT. Because the RCT found no difference in the primary clinical outcomes, a cost minimization analysis was conducted. Process measures of the quality of care such as frequency and length of visits were superior in primary care. Costs to patients and to the health service were lower in primary care. There was no difference in total costs of diagnostic tests, with particular tests being performed more frequently in primary care than in specialist care. Data are provided on the average frequency and length of visits, and frequency of diagnostic testing for breast cancer patients during the follow-up period. © 1999 Cancer Research Campaign


British Journal of Cancer (1999) **79**(7/8), 1227–1233
© 1999 Cancer Research Campaign
Article no. bjoc.1998.0197

### Follow-up of breast cancer in primary care vs specialist care: results of an economic evaluation

**E Grunfeld[1,2,6], A Gray[4], D Mant[1,5], P Yudkin[1], R Adewuyi-Dalton[1], D Coyle[6], D Cole[7], J Stewart[3], R Fitzpatrick[1] and M Vessey[1]**

[1]Division of Public Health and Primary Health Care, Institute of Health Sciences, University of Oxford, Oxford OX3 7LF, UK; [2]Ottawa Regional Cancer Centre, Department of Medicine, University of Ottawa, Ontario K1H 8L6, Canada; [3]Northamptonshire Centre for Oncology, Northampton General Hospital, Northampton NN1 5BD, UK; [4]Health Economics Research Centre, Institute of Health Sciences, University of Oxford, Oxford OX3 7LF, UK; [5]Department of Primary Medical Care, University of Southampton, Southampton SO1 6ST, UK; [6]Clinical Epidemiology Unit, Loeb Research Institute, University of Ottawa, Ontario K1Y 4E9, Canada; [7]Princess Margaret Hospital, Swindon SN1 4JU, UK

**Summary** A randomized controlled trial (RCT) comparing primary-care-centred follow-up of breast cancer patients with the current standard practice of specialist-centred follow-up showed no increase in delay in diagnosing recurrence, and no increase in anxiety or deterioration in health-related quality of life. An economic evaluation of the two schemes of follow-up was conducted concurrent with the RCT. Because the RCT found no difference in the primary clinical outcomes, a cost minimization analysis was conducted. Process measures of the quality of care such as frequency and length of visits were superior in primary care. Costs to patients and to the health service were lower in primary care. There was no difference in total costs of diagnostic tests, with particular tests being performed more frequently in primary care than in specialist care. Data are provided on the average frequency and length of visits, and frequency of diagnostic testing for breast cancer patients during the follow-up period.

**Keywords:** breast cancer; economic evaluation; follow-up care; randomized controlled trial; primary care

After completing primary treatment, it is standard practice in most countries for breast cancer patients to be followed in specialist outpatient clinics during the disease-free interval. This practice of post-treatment surveillance has recently come under close scrutiny (Dewar, 1995; Donegan, 1995; Loprinzi, 1995; Breast Cancer Surveillance Expert Panel, 1997). Studies to evaluate its effectiveness have used a 'less intensive' specialist follow-up regimen as the comparator (GIVIO Investigators, 1994; Rosselli Del Turco et al, 1994). No previous studies have evaluated prospectively the cost effectiveness of this practice of long-term follow-up of breast cancer patients.

We conducted a randomized controlled trial (RCT) comparing specialist follow-up with follow-up by the patient's own general practitioner (Grunfeld et al, 1996). The results showed no increase in delay in diagnosing recurrence and reinitiating specialist care as a result of primary care follow-up. As has been reported in previous studies (Clark and Morris, 1981; Hughes, 1985; Tomin and Donegan, 1987; McWhinney et al, 1990; Worster et al, 1995), the results of the RCT (Grunfeld et al, 1996) showed that most recurrences are detected in the interval between regularly scheduled follow-up appointments, and many are presented to the general practitioner irrespective of the formal follow-up arrangements. The results also showed no increase in anxiety or deterioration in health-related quality of life (HRQOL) (Grunfeld et al, 1996). We report here the results of the economic evaluation to assess the relative costs of the two alternative schemes of follow-up that was conducted concurrent with the RCT. Because the RCT showed no important differences in the primary clinical outcomes, the form of economic evaluation was cost minimization whereby the two schemes are examined for the least costly alternative (Drummond et al, 1997).

*Received 18 May 1998*
*Accepted 30 July 1998*

*Correspondence to:* E Grunfeld, Ottawa Regional Cancer Centre, Department of Medicine, University of Ottawa, 501 Symth Road, Ottawa, Ontario K1H 8L6, Canada

### PARTICIPANTS AND METHODS

### Participants and methods for the RCT

Participants were 296 women with breast cancer in remission receiving regular follow-up at two district general hospitals in England. These women were taking part in an RCT to evaluate a primary-care-based system of routine breast cancer follow-up, whereby they were randomized to one of two groups: continued routine follow-up in hospital outpatient clinics according to usual practice (hospital group) or routine follow-up from their own general practitioner (general practice group). Ethical approval to conduct the study was obtained from the local research ethics committees. The recommended follow-up regimen was the same for women in both groups and involved periodic physician visits for a breast cancer check-up, routine surveillance mammograms (the frequency depended on initial treatment and age), and diagnostic testing only if clinically indicated. A full description of study participants, study methods and results of the primary outcomes of time to diagnosis of recurrence and HRQOL have been reported previously (Grunfeld et al, 1996). Data collection for economic evaluation was an integral part of the RCT.

### Measurement and analysis of costs

The perspective of the economic evaluation considered costs to the health service (particularly, the costs of follow-up visits and diagnostic tests) and costs to the patients (such as lost earnings and out-of-pocket expenses). Valuation of resources was based on unit costs from the providers and national averages. All costs are expressed in 1994 UK £s. Discounting was not considered to be relevant to this analysis, which concerns an 18-month time period.

### *Health service costs*

Information on health service resource use was collected prospectively at both hospital and general practice visits by means of a record-of-visit form. This was completed at all consultations possibly related to breast cancer by doctors for patients in both groups and provided details of the frequency of visits, the duration of each visit, and the diagnostic tests ordered at each visit. The study hospitals provided unit costs for each type of test and for an average outpatient consultation (based on the total annual running and capital costs of the department averaged across the total number of attendances); the equivalent total costs for an average general practice consultation was taken from national averages.

In the analysis of costs, the average of the unit costs for the diagnostic tests, procedures and outpatient consultations provided by the two study hospitals was used.* For estimates of the general practitioner consultations, UK Department of Health estimates of the cost of a consultation were adjusted to allow for the differences in the average length of surgery and home consultations (Wilson, 1991). Using this approach, a cost per minute of £1.15 was estimated. To calculate a cost per GP consultation, the cost per minute was then applied to the length of each consultation.

### *Patient costs*

A questionnaire to collect data on costs incurred by the patient was developed specifically for this study. The cost questionnaire related to costs associated with the patient's most recent breast cancer follow-up visit, and included questions about out-of-pocket expenses, lost earnings as a result of time off work, time spent in attending the follow-up visit, and costs incurred by accompanying persons. When patients, or those accompanying them, indicated that they had lost earnings over a specified period as a result of a follow-up visit, the cost to them of this visit was estimated by applying appropriate national average hourly wage rates†.

The cost questionnaire formed part of the questionnaire package containing the instruments measuring HRQOL, described previously (Grunfeld et al, 1996). The questionnaire package was posted to study participants at three points during the 18-month study period: baseline (before randomization when all follow-up appointments were in hospital outpatient clinics), midtrial and at the end of the trial. One reminder was sent if the questionnaire had not been returned within 2 weeks. Participants completed the midtrial questionnaire within 10 days of a follow-up visit. As there were no important differences in the results obtained at midtrial from those obtained at the end of the trial, midtrial results are reported here because they were more closely linked to a recent follow-up appointment.

*An assurance of confidentiality was requested and given to the study hospitals regarding unit costs. For this reason, only the average of the costs for the study hospitals is given.

†National average wage rates for manual and non-manual labour taken from New Earnings Survey (1994).

### *Analysis*

The two-tailed *t*-test was used to assess the significance of between group differences in means. The $\chi^2$ test was used to assess the significance of between group differences in proportions. Statistical analysis was performed using the software package Statistical Package for the Social Sciences (SPSS, version 6.1.2). Confidence intervals were calculated using the statistical program CIA (Garner, 1991). All analysis was on an intention-to-treat basis.

### Response rates

Completed record-of-visit forms were returned by physicians for 100% (148 out of 148) of patients in the general practice group (i.e. at least one form was received for each patient) and for 95.3% (141 out of 148) of patients in the hospital group.

The baseline cost questionnaire was completed by 99.3% (147 out of 148) of patients in the general practice group and 95.3% (141 out of 148) of patients in the hospital group. By midtrial, four patients had died in the general practice group and five had died in the hospital group. The adjusted response rate for the midtrial cost questionnaire was 97.2% (140 out of 144) and 88.7% (126 out of 142) respectively. At the end of the study period, a total of six patients had died in the general practice group and one had moved and so was lost to follow-up, whereas a total of 12 patients had died in the hospital group and one had moved and so was lost to follow-up. The adjusted response rate for the end of the trial cost questionnaire was 97.2% (137 out of 141) in the general practice group and 88.1% (119 out of 135) in the hospital group.

As the adjusted response rate in the hospital group had fallen to just above 88% while remaining above 97% in the general practice group, non-respondents in the hospital group were compared on baseline characteristics and selected domains of the HRQOL instruments. There were no significant differences between non-respondents and respondents on any of these variables.

### RESULTS

### Health service resource use and costs

During the 18 months of the study, general practice patients were seen significantly more frequently (mean of 3.4 follow up visits) than were specialist patients (mean of 2.8 follow-up visits) (difference 0.6; 95% CI 0.3–0.9; $P < 0.001$). Each follow-up visit was longer in the general practice group (mean 10.5 min) than in the hospital group (mean 7.4 min) (difference 3.1; 95% CI 2.6–3.6; $P < 0.001$), based on physicians' reports on the record-of-visit form. Similarly, over the 18 months, the total time for follow-up visits was longer in the general practice group (mean 35.6 min) than in the hospital group (mean 20.7 min) (difference 14.9; 95% CI 11.3–18.4; $P < 0.001$).

### *Health service costs per patient*

The mean total cost and cost per visit was significantly less per patient in the general practice group than in the hospital group (Table 1). Although there was no difference between groups in the overall cost of diagnostic tests per patient (Table 1), there were significant differences between groups in the costs of individual diagnostic tests (Table 2). General practitioners ordered more of particular diagnostic tests than did specialists, resulting in significantly greater costs for chest radiographs, blood tests [full blood

*© Cancer Research Campaign 1999*

**Table 1** Average costs (£s) per patient by trial group

| Resource item | GP group $n = 148$ Mean (s.d.) | GP group $n = 148$ Range | Hospital group $n = 141$ Mean (s.d.) | Hospital group $n = 141$ Range | Difference GP – Hospital (95% CI) |
|---|---|---|---|---|---|
| Cost of visits | 40.9 (20.1) | 5.8–143.8 | 174.1 (85.1) | 62.0–558.0 | –133.2* (–147.8;–118.7) |
| Cost of tests | 23.8 (29.7) | 0.0–158.2 | 20.9 (36.3) | 0.0–204.9 | 2.9 (–4.8; 10.6) |
| Total costs | 64.7 (42.8) | 5.8–301.9 | 195.1 (107.4) | 62.0–737.4 | –130.4* (–149.1;–111.6) |

*$P < 0.001$.

**Table 2** Mean frequency and cost of each type of test by trial group[a]

| Test | Unit cost (£s)[b] | Mean frequency and costs (£s) GP group No./Pt | GP group Cost/Pt | Hospital group No./Pt | Hospital group Cost/Pt | Difference (95% CI) | *P*-Value |
|---|---|---|---|---|---|---|---|
| Biopsy | 19.52 | 0.02 | 0.39 | 0.021 | 0.42 | –0.001 (–0.039; 0.037) | 0.959 |
| Bonescan | 79.08 | 0.054 | 4.27 | 0.099 | 7.83 | –0.045 (–0.118; 0.028) | 0.223 |
| Chest radiograph | 13.17 | 0.162 | 2.13 | 0.057 | 0.75 | 0.105 (0.029; 0.182) | 0.007 |
| Full blood count | 5.47 | 0.148 | 0.81 | 0.036 | 0.19 | 0.112 (0.042; 0.182) | 0.002 |
| Liver enzymes | 6.84 | 0.108 | 0.74 | 0.021 | 0.14 | 0.087 (0.031; 0.143) | 0.002 |
| Mammograms | 26.37 | 0.493 | 13.00 | 0.355 | 9.36 | 0.138 (0.004; 0.273) | 0.043 |
| Needle biopsy (FNA) | 21.76 | 0.014 | 0.30 | 0.043 | 0.93 | –0.029 (–0.072; 0.014) | 0.188 |
| Abdominal ultrasound | 23.15 | 0.014 | 0.32 | 0.007 | 0.16 | 0.007 (–0.017; 0.03) | 0.592 |
| Radiograph (other) | 20.32 | 0.068 | 1.38 | 0.057 | 1.15 | 0.011 (–0.049; 0.07) | 0.720 |
| ESR | 9.00 | 0.054 | 0.49 | 0 | 0 | 0.054 (0.017; 0.091) | 0.004 |

[a]GP group, $n = 148$; hospital group, $n = 141$; [b]unit costs are the average of the unit costs provided by the study hospitals for each diagnostic test.

count (FBC), erythrocyte sedimentation rate (ESR) and liver enzymes] and mammograms (Table 2).

### *Health service costs per visit*

Over the 18 months of the study, there was a total of 501 follow-up visits in the general practice group and 397 visits in the hospital group. The general practice patients were seen significantly more often than were the specialist patients. Although the mean cost per visit in general practice was significantly less, the mean cost of diagnostic tests per visit was similar in the two groups (Table 3). The lower cost in general practice was attributable to lower physicians' costs per visit (Table 3).

### Patient costs

There were more patients in paid employment in the general practice group than in the hospital group (Table 4). For those patients in paid employment, more in the hospital group took time off work, although there was no difference between groups in the proportion losing wages to attend the follow-up appointment (Table 4). Of note, no more than four patients in either group incurred lost income, and only three patients in the hospital group incurred costs for child care (Table 4). Significantly more patients in the general practice group were able to walk to their appointment (Table 4), and significantly more patients in the hospital group incurred out-of-pocket expenses for car parking (Table 4). With respect to accompanying persons, there were no differences between general practice and hospital group in the proportion who took time off work [4 out of 140 (2.9%) vs 9 out of 126 (7.1%) ($P = 0.21$) respectively] or lost wages [1 out of 140 (0.7%) vs 3 out of 126 (2.4%) ($P = 0.41$) respectively].

Patients in the general practice group spent significantly less time getting to and from their appointment and waiting to see the doctor than patients in the hospital group. Patients in the general practice group, however, spent significantly more time with the doctor than patients in the hospital group (Table 5). For example, based on patients' reports in the cost questionnaire, an average follow-up appointment in the general practice group took a total of 52.6 min (of which 12.8 min were spent with the doctor) compared with 82.2 min (of which 6.9 min were spent with the doctor) in the hospital group (Table 5).

**Table 3** Average cost (£s) per visit by trial group

| Resource item | GP group *n* = 501 Mean (s.d.) | GP group Range | Hospital group *n* = 397 Mean (s.d.) | Hospital group Range | Difference GP – hospital (95% CI) |
|---|---|---|---|---|---|
| Cost of physician | 12.1 (4.6) | 3.5–51.8 | 62.0 | 62.0–62.0 | –49.9* (–50.4;–49.5) |
| Cost of tests | 6.9 (14.8) | 0.0–105.5 | 7.2 (17.8) | 0.0–111.8 | –0.3 (–2.4; 1.9) |
| Total cost per visit | 19.0 (16.6) | 3.5–128.5 | 69.2 (17.8) | 62.0–173.8 | –50.2* (–52.5; –47.9) |

*$P < 0.001$.

**Table 4** Midtrial patient resource events by trial group

| Parameter | GP group *n* = 140[c] No. | GP group % | Hospital group *n* = 126[c] No. | Hospital group % | *P*-Value |
|---|---|---|---|---|---|
| Employed | 65 | 47.8 | 36 | 31.0 | 0.023 |
| Not employed | 71 | 52.2 | 80 | 69.0 | |
| Took time off work | 21 | 32.3 | 22 | 61.1 | 0.006 |
| No time off work | 44 | 67.7 | 14 | 38.9 | |
| Lost wages | 3 | 4.6 | 4 | 11.4 | 0.24 |
| No lost wages | 62 | 95.4 | 31 | 88.6 | |
| Transportation to appointment[a] | | | | | |
| Walk | 45 | 32.4 | 2 | 1.6 | |
| Bus | 11 | 7.9 | 18 | 14.8 | 0.000 |
| Car | 80 | 57.6 | 97 | 79.5 | |
| Other | 3 | 2.2 | 5 | 4.1 | |
| Out-of-pocket expenses | | | | | |
| Yes[b] | 3 | 2.4 | 12 | 11.0 | 0.008 |
| No | 121 | 97.6 | 97 | 89.0 | |
| Need for child care | | | | | |
| Yes | – | – | 3 | 2.6 | 0.06 |
| No | 135 | 100.0 | 114 | 97.4 | |

[a]As the same mode of transportation was used for journey to and from appointment, only data for transportation to appointment shown. [b]In all cases, out-of-pocket expenses were for car parking. [c]For some parameters No. does not sum to '*n*' because of missing data.

**Table 5** Midtrial patient time by trial group

| Parameter | Group | No. | Time (min) Mean (s.d.) | Difference in means (95% CI) |
|---|---|---|---|---|
| Time to get to appointment | GP | 135 | 13.1 (8.3) | –13.56* |
| | Hospital | 120 | 26.7 (15.9) | (–16.65; –10.37) |
| Time to get back from appointment | GP | 138 | 13.6 (8.6) | –14.12* |
| | Hospital | 118 | 27.7 (15.3) | (–17.12; –11.12) |
| Time waiting to see the doctor | GP | 136 | 13.0 (10.7) | –10.3* |
| | Hospital | 119 | 23.3 (19.9) | (–14.2; –6.5) |
| Time with the doctor | GP | 138 | 12.8 (5.8) | 5.9* |
| | Hospital | 121 | 6.91 (4.1) | (4.7; 7.2) |
| | GP | 131 | 52.6 (22.1) | –29.6* |
| Total time for appointment | Hospital | 115 | 82.2 (31.8) | (–36.5; –22.8) |

*$P < 0.001$.

 *© Cancer Research Campaign 1999*

**Table 6** Average costs for 18 months of follow-up by trial group

| | GP group costs (£s) *n* = 148 (average number of visits = 3.39) | Hospital group (£s) *n* = 148 (average number of visits = 2.81) |
|---|---|---|
| Direct medical costs | | |
| Diagnostic tests | 3526.84 | 3100.60 |
| Physician consultations | 6053.20 | 25771.24 |
| Direct non-medical costs[a] | | |
| Patient travel[b] | 817.80 | 748.58 |
| Out-of-pocket expenses[c] | 7.12 | 301.12 |
| Time costs[d] | | |
| Lost wages[e] | | |
| Patient | 62.29 | 107.18 |
| Accompanying person | 20.76 | 80.38 |
| Total | 10488.01 | 30109.10 |

[a]Patient costs based on data from midtrial assessment. [b]Calculation based on reported travel costs to clinics and GP offices uprated to 1994 prices. Average cost to GP office £1.63; average cost to clinic £1.80, assuming approximate proportion of patients driving to GP office is 42% and driving to clinic is 77% (Wilson, 1991). Travel cost per visit multiplied by number of patients and average number of visits per patient in each group [c]Calculation based on out-of-pocket expenses reported by patients at midtrial multiplied by average number of visits per patient in each group. [d]Valuation of leisure time and work time costs (other than lost wages) not included in this analysis. [e]Calculation based on average total time costs of patients at midtrial (gross hourly wage for non-manual labour × average total time for appointment) multiplied by number of patients reporting lost wages (for self or accompanying person) multiplied by average number of visits per patient in each group. Wages based on 1994 gross wages for non-manual employment (almost all patients reporting lost wages were in non-manual labour). (New Earnings Survey, 1994.)

### Cost minimization analysis

The total direct health care and patient costs and time costs for the study cohort during the 18 months of follow-up are reported in Table 6. Despite fewer visits per patient in the hospital group, the large difference in direct medical costs between groups was due to the greater cost of specialist outpatient visits. The difference in direct non-medical costs to patients was due to higher costs incurred by patients in the hospital group for car parking. Patient travel costs (other than car parking) were similar between groups because the larger costs incurred by patients in the hospital group were offset by fewer follow-up visits. Time costs in the form of wages foregone for patients and accompanying persons were greater in the hospital group because of the greater time taken to attend a specialist outpatient visit. This was the case despite similar proportions in each group taking time off work and fewer follow-up visits in the hospital group.

### DISCUSSION

### Health service costs

The calculation of health service costs is based on unit costs for diagnostic tests and outpatient consultations obtained directly from the institutions involved in the study, both of which were district general hospitals in central England. Uncertainty exists as to whether the unit costs provided by the institutions reflect the real costs of the services, and whether they are typical of costs elsewhere in England. The variation in costs in different districts in England (and elsewhere) may result in less cost savings for some institutions and more for others. To facilitate comparison with other settings, data on the quantity as well as the cost of health service resource use are provided (Drummond and Davies, 1991; Drummond and Jefferson, 1996). This study provides data on the average frequency and length of visits, and frequency of diagnostic testing during the routine follow-up period. Hence, this study provides purchasers in district health authorities with the means to calculate for themselves the costs associated with the different follow-up strategies. Although a formal sensitivity analysis was not conducted, even reducing the unit cost of an outpatient consultation by 50% would have minimal impact on the overall findings of the cost analysis.

The cost calculations are based on average costs rather than marginal costs because average costs provide a better estimate of the cost of services affecting a large number of facilities (Drummond and Jefferson, 1996). Thus, the use of average costs enhances the generalizability of the results (Drummond and Jefferson, 1996), but may overestimate the costs in the short-term when compared with a calculation based on marginal costs (Robinson, 1993).

An important finding of this study is that general practitioners ordered more diagnostic tests than specialists. GPs were provided with guidelines that mammograms were the only investigation recommended routinely. Nevertheless, they ordered five times as many FBCs and liver enzymes, three times as many chest radiographs, and eight times as many ESRs. This finding is not surprising because it has been suggested that GPs might order diagnostic tests more frequently than specialists because of lack of confidence in their clinical skills. One other possible explanation is that these tests were ordered because of a co-morbidity for which the GP was also following the patient.

No previous study has compared the cost of specialist follow-up with follow-up in primary care. Other reports have compared ‘intensive’ with ‘minimalist’ follow-up strategies (Schapira, 1993; Mapelli et al, 1995; Virgo et al, 1995). Although the ‘minimalist’ follow-up strategy in those reports is similar to the control arm of this study (periodic examination and routine mammograms), the experimental arm goes one step further by providing ‘minimalist’ follow-up in primary care. The results of the economic evaluation

*© Cancer Research Campaign 1999*

of this study show that the lower cost of the experimental strategy is primarily attributable to the lower cost of a general practitioner consultation compared with a specialist consultation in hospital clinics. This cost saving, however, might be counterbalanced by the documented increased use of diagnostic tests by GPs, and the expected (but not documented) further costs of false-positive test results. Before it can be concluded with certainty that primary care follow-up is the less costly follow-up strategy, further investigation will need to be carried out.

### Patients costs

In this study, data were collected on direct costs to patients (travel, child care, out-of-pocket expenses), time costs (wages lost to patients and accompanying persons), and the time taken for a follow-up visit. Lost wages were valued according to national average wage rates. The time taken for a follow-up visit, however, was significantly greater in the hospital group than in the GP group. In this study, no monetary value was given for the time taken for a follow-up visit. Although it is recognized that there is an opportunity cost associated with time forgone to attend a follow-up visit (Robinson, 1993; Sculpher and Buxton, 1993), valuation of this time was not undertaken because of the unresolved controversy over the best way to value leisure time costs and non-working time costs (Drummond et al, 1997). Further research is warranted to model – according to the different approaches to valuing time – the private time costs associated with the different follow-up strategies. The high prevalence of breast cancer and the length of current follow-up practices suggests that private time costs will have a significant impact when viewed from the societal perspective (Sculpher and Buxton, 1993; Drummond et al, 1997). The finding of this study that significantly more patients walked to general practice visits is consistent with other studies comparing GP and hospital-based screening clinics (Sculpher and Buxton, 1993). Similarly, the finding that a minority of patients in either group took time off work and a very small minority lost wages is consistent with other studies (Sculpher and Buxton, 1993).

In a comparative study of specialist vs. shared primary care/specialist paediatric cancer care, the greatest proportion of saved costs was associated with patient non-medical costs in the form of travel costs, opportunity costs of time and lost wages (Strayer et al, 1980). When one therapy results in better outcomes than another, patients will choose the best therapy regardless of costs. When clinical outcomes are equivalent, however, personal costs will influence patients' decisions about health care alternatives (Strayer et al, 1980). This may be particularly true for screening programmes in which benefits are less tangible and personal costs may prove to be economic barriers to the use of such programmes (Sculpher and Buxton, 1993). The possibility that a proportion of patients may not be attending regular follow-up in specialist clinics because of economic barriers has never been raised in the literature on follow-up. (As noted previously, follow-up can be viewed as a screening programme for recurrent disease.) Although the data from this study cannot illuminate this point (as study patients were already regularly attending for follow-up), it is an interesting possibility that GP follow-up may make the follow-up programme available to a broader range of patients, who may otherwise be deterred by economic barriers.

### CONCLUSIONS

The RCT showed no increase in the clinical outcomes of delay in diagnosing recurrence and reinitiating specialist care as a result of primary care follow-up (Grunfeld et al, 1996). Process measures of the quality of clinical care such as frequency and length of visits (Health Services Research Group, 1992) were superior in primary care.

The cost of physician visits and patient costs were lower in primary care. However, there was no difference in the total costs of diagnostic tests, with particular tests being performed more frequently in primary care than in specialist care. Thus, the lower health service costs of primary care follow-up were attributable to the lower cost of a physician visit. The impact of excess costs of tests and of false-positive test results would have to be evaluated before it can be concluded with certainty that primary care follow-up is the less costly option overall. These excess costs may counterbalance the lower costs of a visit in countries where the cost differential between primary care physicians and specialist physicians is not as great as it is in the UK.

### ACKNOWLEDGEMENTS

We thank Jean Pugh, Jo Horler, Christine Southwell and Sally Black for their excellent and dedicated work as research nurses; Drs John Clements and Graham Cradduck for advice and support in general practitioner liaison; and Karen Lee for data analysis. We thank the many surgeons, oncologists and general practitioners whose commitment made this research possible. We particularly thank the women with breast cancer who participated in this research.

EG is supported in part by the Ontario Ministry of Health. At the time of the research, EG was a fellow of the National Cancer Institute of Canada supported with funds from the Canadian Cancer Society. The research was funded by the Department of Health for England and Wales with a generous contribution from the Ballakermean School of the Isle of Man and support from the General Practice Research Group of the Imperial Cancer Research Fund.

#### REFERENCES

Breast Cancer Surveillance Expert Panel (1997) Recommended breast cancer surveillance guidelines. *J Clin Oncol* **15**: 2149–2156

Clark PB and Morris DL (1981) Management of patients after mastectomy. *Br Med J* **282**: 2095–2096

Dewar J (1995) Follow-up in breast cancer: a suitable case for reappraisal. *Br Med J* **310**: 685–686

Donegan WL (1995) Follow-up after treatment for breast cancer: how much is too much? *J Surg Oncol* **59**: 211–214

Drummond MF and Davies LM (1991) Economic analysis alongside clinical trials. *Int J Technol Assess Health Care* **7**: 561–573

Drummond MF and Jefferson TO (1996) Guidelines for authors and peer reviewers of economic submissions to the BMJ. *Br Med J* **313**: 275–283

Drummond MF, O'Brien B, Stoddart GL and Torrance GW (1997) *Methods for the Economic Evaluation of Health Care Programmes*, 2nd edn. Oxford University Press: Oxford

Garner SB, Winter PD and Garner MJ (1991) CIA statistical program version 1.1

GIVIO Investigators (1994) Impact of follow-up testing on survival and health-related quality of life in breast cancer patients. *JAMA* **271**: 1587–1592

Grunfeld E, Mant D, Yudkin P, Adewuyi-Dalton R, Cole D, Stewart J, Fitzpatrick R and Vessey MPV (1996) Routine follow-up of breast cancer in primary care: a randomised trial. *Br Med J* **313**: 665–669

*© Cancer Research Campaign 1999*

Health Services Research Group (1992) Quality of care. 1. What is quality and how can it be measured? *Can Med Assoc J* **146**: 2153–2158

Hughes LE and Courtney SP (1985) Follow-up of patients with breast cancer. *Br Med J* **290**: 1229–1230

Loprinzi C (1995) Follow-up testing for curatively treated cancer survivors. *JAMA* **273**: 1877–1878

Mapelli V, Dirindin N and Grilli R (1995) Economic evaluation of diagnostic follow-up after primary treatment for breast cancer. *Ann Oncol* **6**: S61–S64

McWhinney IR, Hoddinott SN, Bass MJ, Gay K and Shearer R (1990) Role of the family physician in the care of cancer patients. *Can Fam Phys* **36**: 2183–2186

Robinson R (1993) Costs and cost-minimisation analysis. *Br Med J* **307**: 726–728

Rosselli Del Turco M, Palli D, Cariddi A, Ciatto S, Pacini P and Distante V (1994) Intensive diagnostic follow-up after treatment of primary breast cancer. *JAMA* **271**: 1593–1597

Schapira DV (1993) Breast cancer surveillance – a cost-effective strategy. *Breast Cancer Res Treat* **25**: 107–111

Sculpher MJ and Buxton MJ (1993) *The Private Costs Incurred when Patients Visit Screening Clinics*: *The Cases of Screening for Breast Cancer and for Diabetic Retinopathy*, pp.1–18. Health Economics Group: Brunel University

Strayer F, Kisker CT and Fethke C (1980) Cost-effectiveness of a shared management delivery system for the care of children with cancer. *Pediatrics* **66**: 907–911

Tomin R and Donegan WL (1987) Screening for recurrent breast cancer – its effectiveness and prognostic value. *J Clin Oncol* **5**: 62–67

Virgo KS, Vernava AM, Longo WE, McKirgan LW and Johnson FE (1995) Cost of patient follow-up after potentially curative colorectal cancer treatment. *JAMA* **273**: 1837–1841

Wilson A (1991) Consultation length in general practice: a review. *Br J Gen Pract* **41**: 119–122

Worster A, Wood ML, McWhinney IR and Bass MJ (1995) Who provides follow-up care for patients with breast cancer? *Can Fam Phys* **41**: 1314–1319

*© Cancer Research Campaign 1999*
